# Genomic Insights into Molecular Regulation Mechanisms of Intramuscular Fat Deposition in Chicken

**DOI:** 10.3390/genes14122197

**Published:** 2023-12-10

**Authors:** Yuzhu Cao, Yuxin Xing, Hongbo Guan, Chenglin Ma, Qihui Jia, Weihua Tian, Guoxi Li, Yadong Tian, Xiangtao Kang, Xiaojun Liu, Hong Li

**Affiliations:** 1 College of Animal Science and Technology, Henan Agricultural University, Zhengzhou 450046, China; caoyuzhu9891@163.com (Y.C.); xyx15754@163.com (Y.X.); 18703988707@163.com (H.G.); 13782241532@163.com (C.M.); jqh9702@163.com (Q.J.); tianweihua126@126.com (W.T.); liguoxi0914@126.com (G.L.); ydtian111@163.com (Y.T.); xtkang2001@263.net (X.K.); xjliu2008@hotmail.com (X.L.); 2 International Joint Research Laboratory for Poultry Breeding of Henan, Zhengzhou 450046, China; 3 Henan Key Laboratory for Innovation and Utilization of Chicken Germplasm Resources, Zhengzhou 450046, China

**Keywords:** chicken, intramuscular fat deposition, genetic variation, non-coding RNA, epigenetic modification, estrogen

## Abstract

Intramuscular fat (IMF) plays an important role in the tenderness, water-holding capacity, and flavor of chicken meat, which directly affect meat quality. In recent years, regulatory mechanisms underlying IMF deposition and the development of effective molecular markers have been hot topics in poultry genetic breeding. Therefore, this review focuses on the current understanding of regulatory mechanisms underlying IMF deposition in chickens, which were identified by multiple genomic approaches, including genome-wide association studies, whole transcriptome sequencing, proteome sequencing, single-cell RNA sequencing (scRNA-seq), high-throughput chromosome conformation capture (HiC), DNA methylation sequencing, and m^6^A methylation sequencing. This review comprehensively and systematically describes genetic and epigenetic factors associated with IMF deposition, which provides a fundamental resource for biomarkers of IMF deposition and provides promising applications for genetic improvement of meat quality in chicken.

## 1. Introduction

Intramuscular fat (IMF) content determines meat tenderness, water-holding capacity, and flavor and serves as an important indicator for meat quality evaluation in livestock and poultry [1,2,3]. Intensive selection for poultry based on growth rate and feed efficiency has resulted in lower IMF content and poorer meat quality, which reduces consumer acceptability and preference [4,5,6]. IMF content is a quantitative trait and has a low to moderate estimated heritability of approximately 0.11–0.16 in chickens [7,8]. Despite a lower heritability, IMF still distinctly responds to selection [9,10], indicating that a genetic approach is an effective way to improve IMF deposition in chickens. Hence, it is extremely important to identify the multifaceted molecular mechanisms, develop effective molecular markers of IMF deposition, and thus accelerate the genetic improvement of IMF content in chickens.

IMF deposition is dependent on the proliferation and differentiation of intramuscular preadipocytes, which is complex and orchestrated by synergistic induction of genetic factors, endocrine hormones, environmental effects, etc. [11,12,13,14,15]. With the continuous development and improvement of sequencing technology, new advances have been made in the molecular mechanisms regulating IMF deposition using different genomic approaches. This review aims to summarize the current understanding of regulatory factors responsible for IMF deposition in chickens at transcriptional, post-transcriptional, and epigenetic scales, which not only provide a better understanding of molecular mechanisms underlying IMF deposition but also provide novel insights into the functional biomarkers for improving meat quality in chickens.

## 2. Research Status on the IMF Deposition of Chickens

Chicken IMF content is an important factor that affects meat quality. It is an important selection indicator for poultry breeding, and increasing IMF is an important strategy to improve meat quality. Therefore, resolving the molecular regulation mechanisms of IMF deposition and developing effective molecular markers have been popular research topics in the field of poultry genetic breeding.

### 2.1. Overview of IMF in Chicken

Adipose tissue development is a process consisting of adipogenesis and cellular accumulation of triglyceride (TG) within lipid droplets [16,17]. According to the distribution location, adipose tissue is divided into abdominal fat, subcutaneous fat, and IMF. Chicken IMF is mainly deposited by the proliferation of preadipocytes and an increase in adipocyte size, i.e., the chicken IMF content depends on the number of cells and lipid deposition capacity [18]. In previous studies, IMF was mainly composed of triglycerides, phospholipids, and structural lipids, but the detailed lipid composition of IMF in chickens is unknown [19]. Lipid molecules detected in muscle were classified into four categories: glycerol esters (GLs), glycerophospholipids (GPs), sphingolipids (SPs), and sterolipids (STs) based on the recognized lipid MAPS classification method [20,21].

### 2.2. Candidate Genes That Harbor Expression Correlation with IMF Content

Previous studies revealed the correlations between the expression levels of genes involving lipid metabolism and IMF content. Li et al. (2008) found that compared with Jingxing yellow hens, the expression level of adipocyte fatty acid binding protein 4 (*A-FABP*) was significantly increased in Beijing You hens, which have higher IMF content, and *A-FABP* expression was significantly higher in males than in females [22]. Zhang et al. (2017) and Zhang et al. (2018) found that the expression levels of adiponectin receptor 1/2 (*ADIPOR1/2*) and fibroblast growth factor 1/10 (*FGF1/10*) were positively correlated with IMF content in the leg muscles of male chickens, whereas other studies on IMF deposition in Tibetan chickens showed that the mRNA levels of *FGF1* and *FGF10* were negatively correlated with IMF content in female chickens [23,24]. Li et al. (2016) found that the expression level of the secreted frizzled-related protein 5 gene (*SFRP5*) in the pectoral and leg muscles of Tibetan chickens correlated with IMF content [25]. Sun et al. (2019) found that Krüppel-like transcriptional factor 9 (*KLF9*) is involved in regulating chicken intramuscular preadipocyte differentiation [26]. These findings indicate that chicken IMF deposition is influenced by various factors, such as breed, sex, and developmental period.

### 2.3. Identification of Candidate Genes Controlling IMF Deposition Using Genome-Wide Association Study

IMF content belongs to a quantitative trait that is regulated by multiple genes. Genome-wide association study (GWAS) is a powerful tool to identify genetic variation associated with IMF content at the genomic level, including single nucleotide polymorphism (SNP), insertion/deletion (InDel), copy number variation (CNV), and quantitative trait loci (QTL) [27]. According to the chicken QTL database (Release 50, 25 April 2023), there are three QTLs associated with IMF percentage. For example, Nassar et al. (2013) localized a QTL for IMF content at GGA14 by genomic scanning of 278 F_2_ roosters produced from crosses between New Hampshire and White Leghorn chickens [28]. TYRO3 protein tyrosine kinase (*TYRO3*), microsomal glutathione S-transferase 1 (*MGST1*), kinesin family member 2A (*KIF2A*), and nucleoside-triphosphatase cancer-related (*NTPCR*) were identified as genes potentially related to IMF content in the pectoral muscle of the F_2_ resource population of Beijing You chicken and commercial fast Cobb Vantress [29]. Liu et al. (2013) identified genomic regions that influence IMF content in pectoral muscle by GWAS on meat quality traits in Beijing oil chickens and identified two potentially related genes, cholecystokinin (*CCK*) and toll-interacting protein (*TOLLIP*) [30]. Based on the genetic structure analysis of the Jingxing yellow-feathered chicken population selected for high IMF content differentiation in generations 15 and 16, genes and pathways related to IMF were identified, including acyl CoA synthase long chain family member 1 (*ACSL1*), acyl CoA dehydrogenase long chain (*ACADL*), phospholipid phosphatase 3 (*PLPP3*), membrane-bound O-acyltransferase domain 1 (*MBOAT1*), fatty acid binding proteins 6 and 7 (*FABP6* and *FABP7*), aldehyde dehydrogenase 3 family member A2 (*ALDH3A2*), peroxisome proliferator-activated receptor (*PPAR*), the PPAR signaling pathway, the glycerolipid metabolic pathway, and the fatty acid degradation pathways [8]. In addition, the TG content in muscle largely reflects IMF content [31]. A GWAS for TG content in 520 selected populations with high IMF content demonstrated that the solute carrier family 16 member 7 gene (*SLC16A7*) can promote TG deposition by regulating de novo lipogenesis and is an important candidate gene responsible for TG content in chicken muscle tissue [32].

Ye et al. (2010) found that SNPs occurring in the *A-FABP* and *H-FABP* genes in male Beijing oil chickens had a significant effect on IMF (*p* < 0.05), with chickens with the BB genotype at the *A-FABP* gene having significantly higher IMF than those of the AA and AB genotypes, and chickens with the DD and CD genotypes at the *H-FABP* gene having much higher IMF than those of the CC genotype [33]. Shu et al. (2015) found that SNP (CAPN1 3535) of the *CAPN1* gene was significantly associated with meat quality traits, with higher IMF content in individuals carrying the AA genotype [34]. Cui et al. (2018) found that two SNPs (G7518A and C7542G) occurring in intron 4 of the calpain 9 (*CAPN9*) gene (GenBank accession No. XM_419585) were significantly associated with pectoral muscle percentage (*p* < 0.05), and the AA (G7518A) genotype and GG (C7542G) genotype had the highest IMF content, highest pectoral muscle weight, and lower abdominal fat weight (AFW) and abdominal fat percentage (AFP) [35]. Wang et al. (2022) demonstrated that the SNP rs17631638T>C in the ELOVL3 promoter was significantly correlated with IMF through affecting its expression, and *ELOVL3* may promote fat deposition in muscles by increasing the proportion of long-chain unsaturated glycerol phospholipid molecules in the breast muscle [36]. Wang et al. (2023) found that C12315T, a synonymous mutation in the lipoprotein lipase (*LPL*) gene (XM_015280414.2), was significantly positively correlated with IMF in the pectoral and leg muscles of Baicheng-You chicken and San-huang chickens [37]. These data indicate that IMF deposition is often subject to complex regulation of multiple loci (genes).

### 2.4. Identification of Candidate Genes Controlling IMF Deposition at the mRNA Level 

The transcriptomics is widely acknowledged as an effective method to directly identify candidate genes and regulatory mechanisms associated with IMF deposition at the genomic level (Table 1). Cui et al. (2012) identified that 3-hydroxymethyl-3-methylglutaryl-CoA lyase like 1 (*HMGCLL1*), thrombospondin 1 (*THBS1*), uncoupling protein 3 (*UCP3*), enoyl-CoA hydratase and 3-hydroxyacyl CoA dehydrogenase (*EHHADH*), and sorting nexin 4 (*SNX4*) were potential candidate genes that were associated with IMF deposition by comparative analysis of the pectoral muscle transcriptomes of slow-growing Peking oil chickens and faster-growing commercial AA broilers [38]. Ye et al. (2014) identified potential candidate genes associated with IMF deposition by comparative transcriptome analysis of the leg muscles of 7-week-old normal and sex-linked dwarf chickens, and they found that IMF deposition in the leg muscles of sex-linked dwarf chickens is partially regulated by adipocytokines, insulin, and other downstream signaling pathways (TGF-β/SMAD3 and Wnt/catenin-β pathways) [39]. Qiu et al. (2017) revealed that down-regulation of solute carrier family 27 member 1 (*SLC27A1*), also known as fatty acid transport 1 (*FATP1*), could reduce CPT1A-mediated fatty acid oxidation and thus promote IMF deposition in chickens [40]. 

Pectoral and leg muscles constitute an overwhelming majority of meat production in chickens, and studies have shown that the IMF content in the leg muscle is significantly higher than that in the thoracic muscle [38], but the regulatory mechanism is unclear. Cui et al. (2018) analyzed differentially expressed genes in the pectoral and leg muscles of 42- and 90-day-old Beijing You chickens and showed that peroxisome proliferator activated receptor γ (*PPARG*) and its downstream genes have important regulatory functions for IMF deposition [41]. 

As the main component of the IMF, TG largely reflects the IMF content and therefore plays an important role in IMF deposition [31,42]. Comparative transcriptome analysis of pectoral muscle tissues from Jingxing yellow chickens with divergent intramuscular TG content demonstrated that, the expression levels of key genes involved in lipid synthesis, such as adiponectin, C1Q and collagen domain containing (*ADIPOQ*), cluster of differentiation 36 (*CD36*), *FABP4*, *FABP5*, cell death-inducing DNA fragmentation factor-like effector C (*CIDEC*), *LPL*, stearoyl-CoAdesaturase (*SCD*), perilipin 1 (*PLIN1*), and *PPARG*, and genes involving in steroid biosynthesis, such as 24-Dehydrocholesterol Reductase (*DHCR24*), Methylsterol Monooxygenase 1 (*MSMO1*), lanosterol synthase (*LSS*), NAD (P) Dependent Steroid Dehydrogenase-Like (*NSDHL*) and cholesterol 25-Hydroxylase (*CH25H*) were significantly higher in the high TG group than in the low TG group; additionally, the steroid biosynthesis and PPAR signaling pathway played a key role in IMF deposition [43]. 

Transcriptome analysis of pectoral muscles from Beijing You chickens at different developmental periods revealed that genes related to energy metabolism, such as acyl-CoA thioesterase 9 (*ACOT9*), cholesteryl ester transfer protein (*CETP*), *LPIN1*, diacylglycerol O-acyltrasferase 2 (*DGAT2*), retinol binding protein 7 (*RBP7*), fructose-bisphosphatase 1 (*FBP1*), and phosphorylase kinase regulatory subunit α 1 (*PHKA1*), could regulate IMF deposition [44]. Weighted co-expression network analysis of the transcriptomes of Wenchang chicken pectoral muscle tissue found that IMF deposition in pectoral muscle was associated with glyceraldehyde-3-phosphate dehydrogenase (*GAPDH*), lactate dehydrogenase A (*LDHA*), glutathione peroxidase 1 (*GPX1*), and 1,4-α-glucan branching enzyme 1 (*GBE1*), which are involved in pyruvate and citrate metabolism, and carbohydrate metabolism exerted an important role in IMF deposition [45]. Transcriptomic analysis of the leg muscle of 60-day-old caged and free-range Luyang woolly bone chickens revealed that IMF deposition may be associated with upregulated expression of genes involved in the PPAR signaling pathway, including angiopoietin-like 4 (*ANGPTL4*), *CD36*, fatty acid transport proteins 1 and 4 (*FATP1*, *FATP4*), and perilipin 2 (*PLIN2*) [46]. The binding of glucocorticoid to its receptors may modulate the ADPNR-PPARα-FATP1 pathway, thereby regulating the uptake of saturated fatty acids by myocytes [47]. Comparative transcriptomes of the pectoral and leg muscles of Zhuanghe Dagu chickens and AA broilers demonstrated that the extracellular matrix-receptor interaction pathway regulates IMF deposition by affecting the metabolism of intermuscular adipocytes [48]. Based on the transcriptome analysis of the pectoral muscle tissues of female Jingxing yellow chickens from 12 embryonic to 180 days of age, the hub gene such as ENSGALG00000041996, transcript factor l (3) mbt-like 1 (*L3MBTL1*), and the transcription factor cofactors TNFAIP3 interacting protein 1 (*TNIP1*), histone acetyltransferase 1 (*HAT1*), and BEN domain containing 6 (*BEND6*) were identified as being associated with high breast muscle IMF [49].

**Table 1:** Candidate genes controlling IMF deposition reported by more than two studies.

Gene and Pathway	Function	Reference
PPAR pathway	Regulate IMF deposition.	[8,43,46,50,51,52,53,54]
*FATP1*	Regulating the uptake of saturated fatty acids into myoblasts reduces CPT1A-mediated fatty acid oxidation and thus promotes IMF deposition in chickens.	[40,47,48,55]
*FASN*, *SREBP1*, *SCD*	Involving lipid synthesis	[11,43,56,57]
*PPARG*	Promoting intramuscular adipocyte differentiation	[41,43,58]
*H-FABP*, *A-FABP*	Positively associated with IMF content and influenced by chicken gender	[22,33,59,60]
*ELOVL3*	Promote fat deposition in muscles by increasing the proportion of long-chain unsaturated glycerol phospholipid molecules in the breast muscle.	[36,60]
*PLIN2*	Promote IMF deposition.	[46,61]
*APOA1*	Potential biomarkers for IMF	[57,62]

### 2.5. Non-Coding RNAs Controlling IMF Deposition

Non-coding RNAs, such as circular RNA (circRNA), microRNA (miRNA), and long non-coding RNA (lncRNA), function as endogenous regulators in lipid metabolism. Increasing evidence has suggested the association of non-coding RNAs with IMF deposition in chickens [63,64]. Fu et al. (2018) constructed the first dynamic miRNA expression profile of Gushi chicken pectoral muscle tissue at 6, 14, 22, and 30 weeks of age by whole transcriptome analysis and found that miRNAs such as *miR-138-2-3p*, *miR-103-3p* [65], and *miR-15a* [66] play key roles in IMF deposition by integration analysis with the mRNA transcriptome. Li et al. (2019) used weighted gene co-expression network analysis to identify six gene expression modules that were significantly and positively associated with IMF traits and identified key candidate genes that influence IMF deposition [67]. *Gga-miR-140-5p* promoted intramuscular adipocyte differentiation by targeting retinoid X receptor γ (*RXRG*) [68]. *MiRNA-223* regulates interstitial adipocyte differentiation by targeting *GPAM* [69]. Li et al. (2019) showed that *miR-15a* promotes intramuscular preadipocyte differentiation, increases cholesterol and TG accumulation in adipocytes, and regulates intramuscular fat deposition by targeting sterol carrier protein 2 (*SCP2*), acetyl-CoA acyltransferase 1 (*ACAA1*), and acyl-CoA oxidase 1 (*ACOX1*) in chickens [66]. Sun et al. (2019) performed miRNA-seq by constructing an intramuscular adipocyte differentiation model and found that *gga-miR-18b-3p* inhibited chicken intramuscular adipocyte differentiation by targeting acyl-CoA thioesterase 13 (*ACOT13*) [70]. Lin et al. (2022) found that *miRNA-24-3p* dominated IMF deposition in chickens by promoting the proliferation and inhibiting the differentiation of intramuscular preadipocytes through blocking the expression of membrane-associated protein A6 (*ANXA6*) [71]. Gai et al. (2023) showed that the IMF content of Beijing-You Chicken (BJY) at 1 d of age was significantly higher than that at later stages of birth, and *miR-6701-3p-PTEN*, *miR-1563-WWP1*, *miR-6701-3p-BMPR1B*, *miR-29c-3p-PIK3R1*, and *miR-449c/d-5p-TRAF6* were identified as the key mRNA-miRNA pairs for regulating IMF deposition [72]. Zhu et al. (2023) found that *miR-128-3p* inhibited chicken intramuscular adipocyte differentiation through down-regulation of farnesyl diphosphate synthase (*FDPS*) [73]. Guo et al. (2023) found that LncHLEF can not only act as a molecular sponge to adsorb *miR-2188-3p* to weaken the inhibitory effect on *GATA6*, but also promote hepatic lipid synthesis through its encoded micropeptide and regulate liver-derived secretion. Increased IMF deposition in chicken muscle is mediated by the body [74].

**Figure 1:**
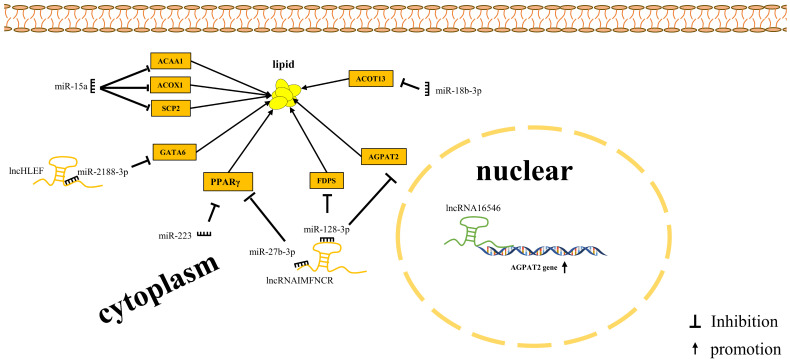
Regulatory map of intramuscular fat deposition by miRNAs and lncRNAs. *ACAA1*, acetyl-CoA acyltransferase 1; *ACOX1*, acyl-CoA oxidase 1; *SCP2*, sterol carrier protein 2; *GATA6*, GATA binding protein 6; *PPARγ*, peroxisome proliferator activated receptor γ; *AGPAT2*, 1-acylglycerol-3-phosphate-O-acyltransferase 2; *FDPS*, farnesyl diphosphate synthase; *ACOT13*, acyl-CoA thioesterase 13.

Although a set of miRNAs and miRNA-mRNA regulatory networks have been identified in chicken muscle tissue and intramuscular adipocyte cells, their specific effects on IMF deposition are poorly understood. To date, only a few miRNAs have been functionally characterized in intramuscular adipogenesis in chickens (Figure 1). Zhang et al. (2018) found that lncRNA *IMFNCR* adsorbs *miR-128-3p* and *miR-27b-3p* through the ceRNA mechanism to enhance PPARG expression, thereby promoting intramuscular adipocyte differentiation [58]. Comparative transcriptome analysis of chicken pectoral muscles with high and low IMF content indicated that lncRNA may regulate IMF deposition by mitogen-activated protein kinase (MAPK), PPAR, gonadotropin-releasing hormone (GnRH), erythroblastic leukemia viral oncogene homolog (ErbB), and calcium signaling pathways, as well as fatty acid elongation and fatty acid metabolism signaling pathways [50]. The dynamics of lncRNA and mRNA expression profiles in the pectoral and leg muscles between Rose Crown and Cobb broiler embryos displayed that a differentially expressed lncRNA, *lncRNA-46546*, significantly increased the expression of 1-acylglycerol-3-phosphate-O-acyltransferase 2 (*AGPAT2*) and lipid droplet accumulation in chicken intramuscular preadipocytes, which might promote IMF deposition in chickens [75]. These studies demonstrated that miRNA, lncRNA, and circRNAs are important regulators of IMF deposition in chickens. Zhang et al. (2020) integrated the RNA-seq and miRNA-seq data of chicken intramuscular adipose tissue and identified that *circARMH1*, *circLCLAT1*, *circFNDC3AL*, and *circCLEC19A* potentially modulate adipogenesis by regulating miRNAs through PPAR and fatty acid metabolic pathways [51]. However, most studies have mainly focused on miRNAs, and fewer studies have reported on lncRNAs and circRNAs in regulating IMF deposition.

### 2.6. Epigenetic Modificators Controlling IMF Deposition 

N-6 methyl adenine (m^6^A) is an important epigenetic modification of RNA in eukaryotic genomes that affects various biological processes [76,77,78,79]. Methylation is also an important epistatic regulator of gene expression [52]. Therefore, changes in methylation levels of genes related to lipid metabolism may also affect IMF deposition. 

Fat mass and obesity-associated (FTO) protein acts as a demethylase, encoded by the *FTO* gene, and was found to regulate adipocyte development. Zhang et al. (2016) found that *FTO* could promote triglyceride lipid deposition in chicken liver by targeting the removal of m^6^A modification of the fatty acid metabolism gene carnitine palmitoyltransferase 1A (*CPT1*) in a model of lipopolysaccharide (LPS)-induced hepatic TG accumulation [80]. Hu et al. (2020) found that hepatic FTO could be activated by cortisol, which promotes hepatic lipid deposition by activating lipogenic genes (*SREBP1*, *FASN*, *ACACA*, and *SCD*) through the effect of m^6^A-modified demethylation [56]. Feng et al. (2021) constructed a glucocorticoid receptor (GR)-mediated corticosterone-induced fatty liver syndrome (FLS) model in chickens and found that hepatic lipid accumulation was increased in laying hens fed a low-protein (HELP) diet (*p* < 0.05), which may be caused by specific clearance of m^6^A modifications of *FASN*, *SREBP1*, and *SCD* lipogenesis-related genes [81]. Li et al. (2022) used different doses of methyl donor betaine and methylation inhibitor cycloleucine to treat chicken primary preadipocytes and found that m^6^A modification was negatively correlated with chicken preadipocyte production and *FTO* through demethylation. Chemically regulates the expression of *CTNNBI*, thereby promoting adipogenesis [82].

Zhang et al. (2023) conducted RNA immunoprecipitation sequencing (MeRIP-seq) and RNA sequencing (RNA-seq) analyses on breast and leg tissues from 180-day-old Jingyuan chickens, revealing differentially methylated genes involved in the regulation of muscle lipid anabolism, including enoyl-CoA hydratase 1 (*ECH1*), branched chain amino acid transaminase 1 (*BCAT1*), and cytochrome P450 family 1 subfamily B member 1 (*CYP1B1*) [83]. Yu et al. (2023) demonstrated that the m^6^A-induced ferroptosis pathway in breast muscle tissue was a novel target for regulating IMF metabolism and validated the finding that chicken Leiomodin 2 (*LMOD2*) and its multiple m^6^A negative regulatory DMGs are potential regulators of differential IMF deposition in muscle [84]. These provided an important theoretical basis for exploring the functional mechanism of m^6^A in IMF deposition in chickens. Zhang et al. (2017) analyzed genome-wide methylation levels in pectoral muscle tissue at different developmental periods and found that high IMF levels were associated with downregulation of transcript levels caused by promoter hypermethylation of ATP binding cassette subfamily A member 1 (*ABCA1*), collagen type VI α 1 chain (*COL6A1*), and glutathione S-transferase theta 1-like (*GSTT1L*) [85]. These studies demonstrated that many different genes regulate chicken IMF deposition, among which the PPAR signaling pathway may play an important role, but the complex molecular mechanisms are not yet clear.

### 2.7. Proteomic and Other Histological Research

Previous studies on the mechanisms of IMF deposition in chickens have mainly focused on the genomic and transcriptomic levels. Technologies such as proteomics, metabolomics, scRNA-seq, and HiC have been developed and gradually applied to elucidate the molecular mechanisms underlying IMF deposition in chicken. A first study linking proteomics to IMF deposition shows that Liu et al. (2016) used iTRAQ technology to analyze the protein expression profiles of the postnatal thoracic muscles of Beijing-You chickens at different developmental stages and identified that apolipoprotein A1 (*APOA1*) and heat shock protein family B (small) member 1 (*HSPB1*) were potential biomarkers of IMF deposition [57]. Then, Liu et al. (2017) quantified the proteome and metabolome of breast muscle of Beijing You chickens and Cobalt broilers at different ages using mass spectrometry and found that protein processing and PPAR signaling pathways promote IMF deposition [53]. Tian et al. (2021) used Hi-C technology to study the effect of chromatin interaction on IMF deposition in the breast muscles of local Chinese chickens and fast-growing AA broilers and found that 3-hydroxy-3-methylglutaryl-CoA reductase (*HMGCR*), regulated by TAD boundary slip, was a potential biomarker for IMF deposition in the breast muscles of chickens [86]. A scRNA-seq analysis of chicken breast muscles identified the *APOA1* and collagen type I α 1 chain (*COL1A1*) genes as biomarkers for chicken IMF cells [62]. 

### 2.8. Hormonal Regulation of Intramuscular Fat Deposition in Chickens

Estrogen is generally considered an important regulator of lipid metabolism. Li et al. (2021) found that the IMF content was significantly higher in females than in males of 150-day-old Daheng broilers, probably due to the differential expression of some genes related to lipid metabolism in the muscle and liver of males and females, and that *PLIN2* may play a key role [61]. Another important aspect is the existence of interactions between intramuscular preadipocytes and skeletal muscle satellite cells that are essential for postnatal skeletal muscle growth and regeneration. Guo et al. (2021) investigated the effect of intramuscular preadipocytes on chicken myosatellite cells by transcriptome analysis and elucidated that intramuscular preadipocytes may promote their lipid deposition through the PPAR pathway [54]. Liu et al. (2017) showed that additional energy from the yolk sac is transported and deposited as IMF in the pectoralis major muscle of chickens at hatching and that lipid metabolism-related genes and pathways (e.g., TGF-β, PPAR, Hedgehog, and cytokine-cytokine receptor interaction signaling pathways) promote IMF deposition in the pectoralis muscle of fast-growing chickens compared with slow-growing chickens [52]. Insulin, a secreted hormone in chickens, can affect the accumulation of body fat in chickens by influencing the expression of genes related to lipid metabolism [87]. Related studies have shown that insulin stimulates the expression of adipogenesis-related genes such as *SCD*, *FASN,* and *LPL* [11,88].

## 3. Conclusions

Many important candidates, including genes, transcription factors, ncRNAs, and epigenetic modification factors, have been identified in the regulation of chicken IMF, which provides a theoretical basis for genetic breeding of high meat quality in chicken. The PPAR pathway is the key signaling pathway for regulating IMF deposition. *FATP1* promotes IMF deposition in chickens by reducing CPT1A-mediated fatty acid oxidation or involving the ADPNR-PPARα-FATP1 pathway to regulate the uptake of saturated fatty acids into myoblasts. *ELOVL3* contributes to increasing the proportion of long-chain unsaturated glycerol phospholipid molecules in the breast muscle. *MiR-15a*, *miR-18b-3p*, *miR-223*, *miR27b-3p*, and *miR-128-3p* were shown to regulate IMF deposition by directly targeting lipid metabolism-related genes and intramuscular adipocyte differentiation-related genes in chickens. LncRNA *IMFNCR* promotes intramuscular adipocyte differentiation by sponge-adsorbing *miR-128-3p* and *miR-27b-3p* to facilitate *PPARG* expression. *LncHLEF* can not only act as a molecular sponge to adsorb *miR-2188-3p* to weaken the inhibitory effect on *GATA6*, but also promote hepatic lipid synthesis through its encoded micropeptide and regulate liver-derived secretion. Increased IMF deposition in chicken muscle is mediated by the body. DNA methylation controls the expression level of *COL6A1* to affect IMF deposition. All these findings show that the formation of IMF is a complex and finely controlled process regulated by multiple genetic/epigenetic factors. Ultimately, the findings of the review can provide novel insights into the DNA, mRNA, protein, non-coding RNA, epigenetic modifiers, and hormone-level regulatory mechanisms of IMF deposition in chickens. Additionally, these results may facilitate the identification of functional SNPs, genes, or epigenetic factors as genetic markers for improving the quality of chicken meat in genetic breeding.

## Data Availability

Not applicable.
